# The STAT1 lexicon: translating signaling codes in *H. pylori*-driven gastric carcinogenesis

**DOI:** 10.3389/fimmu.2026.1679212

**Published:** 2026-10-22

**Authors:** Wei Han, Hongzheng He, Bei Ye, Yilin Sun, XiuPing Wang, Tingting Liu, Hanwei Cui, Jianjun Wang

**Affiliations:** 1 Science and Education Department, Kunshan Maternity and Children’s Health Care Hospital, Suzhou, Jiangsu, China; 2 Department of General Surgery, Kunshan Hospital Affiliated to Jiangsu University, Suzhou, Jiangsu, China; 3 Department of Clinical Laboratory, Jinxi People’s Hospital of Kunshan, Suzhou, Jiangsu, China; 4 Department of Clinical Laboratory, Kunshan Hospital Affiliated to Jiangsu University, Suzhou, Jiangsu, China; 5 Science and Technology Talent Department, Kunshan Hospital Affiliated to Jiangsu University, Suzhou, Jiangsu, China; 6 Central Laboratory, The Fourth People’s Hospital of Shenzhen, Shenzhen, Guangdong, China

**Keywords:** biomarker, gastric cancer, *H. pylori*, immunotherapy, interferons, STAT1, tumorigenesis, tumor microenvironment

## Abstract

Helicobacter pylori (*H. pylori*) is a chronic gastric mucosa colonizer that leads to the Correa cascade of gastric adenocarcinoma progression: chronic gastritis, intestinal metaplasia, and dysplasia. Signal transducer and activator of transcription 1 (STAT1) is one of the key mediators at the host immunity-tumor cell interface. The interaction of *H. pylori* with pattern recognition receptors on gastric epithelial cells induces tyrosine phosphorylation of STAT1 by Janus kinase (JAK), which in turn leads to dimerization and nuclear translocation of the protein, resulting in the suppression of pathogen survival enabled by interferon (IFN)-regulatory transcriptional programs. Ironically, STAT1 has a dual role in gastric carcinogenesis in a context-dependent manner: in the early disease, it acts as a tumor suppressor by facilitating apoptosis and cell-cycle arrest in gastric lesions, but in the advanced disease, it plays a role in immune evasion and tumor progression through the expression of programed death-ligand 1 (PD-L1) and tumor microenvironment (TME) modulation. This review critically assesses how *H. pylori* manipulates the STAT1 signaling during the infection process, crosstalk between the STAT1 and STAT3 pathways, and the future use of this understanding to therapeutically target and develop biomarkers. New strategies such as small-molecule STAT inhibitors, combination immunotherapy, and artificial intelligence (AI)-based biomarker discovery are discussed, as well as existing limitations and future research directions.

## 1 Introduction

Helicobacter pylori (*H. pylori*) is a Gram-negative, microaerobic bacterium that is one of the predominant members of the gastric microbiota. It has been estimated that around 43.1 percent of the world population is infected with *H. pylori*, with significant regional variations: *H. pylori* infection is found in more than 70 percent of the population in some parts of Africa and South America, and less than 30 percent in Northern Europe and North America (1). The International Agency for Research on Cancer (IARC) has classified *H. pylori* as a Group I carcinogen (2, 3). Epidemiological and clinical studies have established that *H. pylori* infection significantly increases the risk of developing duodenal ulcers, gastric ulcers, non-cardia gastric adenocarcinoma, and B-cell malignancies, including gastric mucosa-associated lymphoid tissue (MALT) lymphomas (4, 5). Virtually all infected individuals develop chronic gastritis, which may progress to peptic ulcer disease, atrophic gastritis, and gastric cancer (GC) (6, 7).

Importantly, the pathogenesis of *H. pylori*-associated GC adheres to the well-characterized Correa cascade, which is a multistep process involving the progressive progression of normal gastric mucosa to chronic non-atrophic gastritis, multifocal atrophic gastritis, intestinal metaplasia (IM), dysplasia, and eventually invasive gastric adenocarcinoma (8). On the molecular scale, this cascade is precipitated by persistent disproportion between gastric epithelial cell division and cell demise (9, 10). *H. pylori* has a range of virulence factors that mainly mediate the gastrointestinal pathology due to cytotoxin-associated gene A (CagA) and vacuolating cytotoxin A (VacA), which stimulate tissue-damaging signaling cascades (4, 11, 12). CagA is a bacterial oncoprotein; when injected into host cells by the type IV secretion system (T4SS), CagA is tyrosine phosphorylated and activates Src homology 2 domain-containing protein tyrosine phosphatase 2 (SHP2), thus activating the rat sarcoma-extracellular regulated protein kinases (Ras-ERK pathway), which causes cell proliferation, differentiation, and survival (13–15). Concurrently, CagA and VacA elicit dysregulated immune responses, highlighting the complex interplay between pathogen virulence and host immune surveillance during persistent infection (16). *H. pylori* infection triggers activation of both innate and adaptive immunity (17, 18).

This is followed by an inflammatory response, which recruits macrophages, neutrophils, lymphocytes, dendritic cells, and fibroblasts. Macrophages are developed out of monocytes that circulate in the blood and play key roles in both innate and adaptive immunity (19, 20). *H. pylori* infection triggers the activation of the classical activated (M1) macrophages to secrete reactive oxygen species (ROS), nitric oxide (NO), and pro-inflammatory cytokines, such as interleukin-6 (IL-6), IL-8, IL-23, and tumor necrosis factor-α (TNF-α), which have bactericidal and tumoricidal effects (21–23). But irony has it that chronic infection leads to macrophage polarization towards an alternatively activated (M2) phenotype in the TME, thus fostering tumor initiation and progression (20, 23). Subsequent dysregulated immune response and persistent presence of an inflammatory cytokine environment contribute to *H. pylori*-related gastric pathology and increase the risk of GC (8, 24–26).

Signal transducer and activator of transcription 1 (STAT1) is an important transcription factor that is of paramount importance in the host immune response to the infection of *H. pylori*. When activated, STAT1 facilitates IFN-dependent signaling, which coordinates the expression of antimicrobial genes and production of inflammatory cytokines (27). On the other hand, *H. pylori* has developed advanced immune evasion measures such as inhibition of STAT1 phosphorylation through activation of SHP2, which silences IFN-7 signaling and enables chronic colonization (28). Therefore, despite its importance in controlling the host immune response, an abnormal control of STAT1 in chronic infection can paradoxically enhance carcinogenesis mediated by inflammation (29). Modulation of the activity of STAT1 can be a good way to treat gastric diseases caused by *H. pylori*.

## 2 The biological functions of STAT1

STAT1 was the first member of the STAT protein family to be identified and characterized for its role in IFN signaling (30). The STAT1 gene, located at chromosome 2q32.2, contains 25 exons and undergoes alternative splicing to produce two major isoforms: full-length STAT1α (750 amino acids) and truncated STAT1β (712 amino acids) (**Figure 1**) (31). STAT1α contains critical phosphorylation sites at tyrosine 701 (Y701) and serine 727 (S727) within its C-terminal transactivation domain (TAD); although STAT1β lacks the distal portion of the TAD, it retains the capacity for efficient Y701 phosphorylation (**Figure 1**) (31). STAT1 is an inactive monomer that is located in the cytoplasm in its resting state and has been studied over the last decade with respect to cancer biology (32). STAT1 is activated by a diverse array of extracellular stimuli, including type I IFNs (IFN-α and IFN-β), type II IFN (IFN-γ), type III IFNs (IFN-λ1–λ4), cytokines (IL-6, IL-27, TGF-β1), and growth factors (**Table 1**) (33, 34). The JAK/STAT1 signaling cascade is a canonical pathway activated by IFNs binding to their respective receptors and resulting in the activation of receptor-bound JAK and phosphorylation of the receptor intracellular domain (35–37). Tyrosines in phosphorylated receptor tyrosines are docking sites of STAT1 SH2 domains, which allow the recruitment of STAT1 and JAK-dependent phosphorylation at Y701 (38, 39).

**Figure 1:**
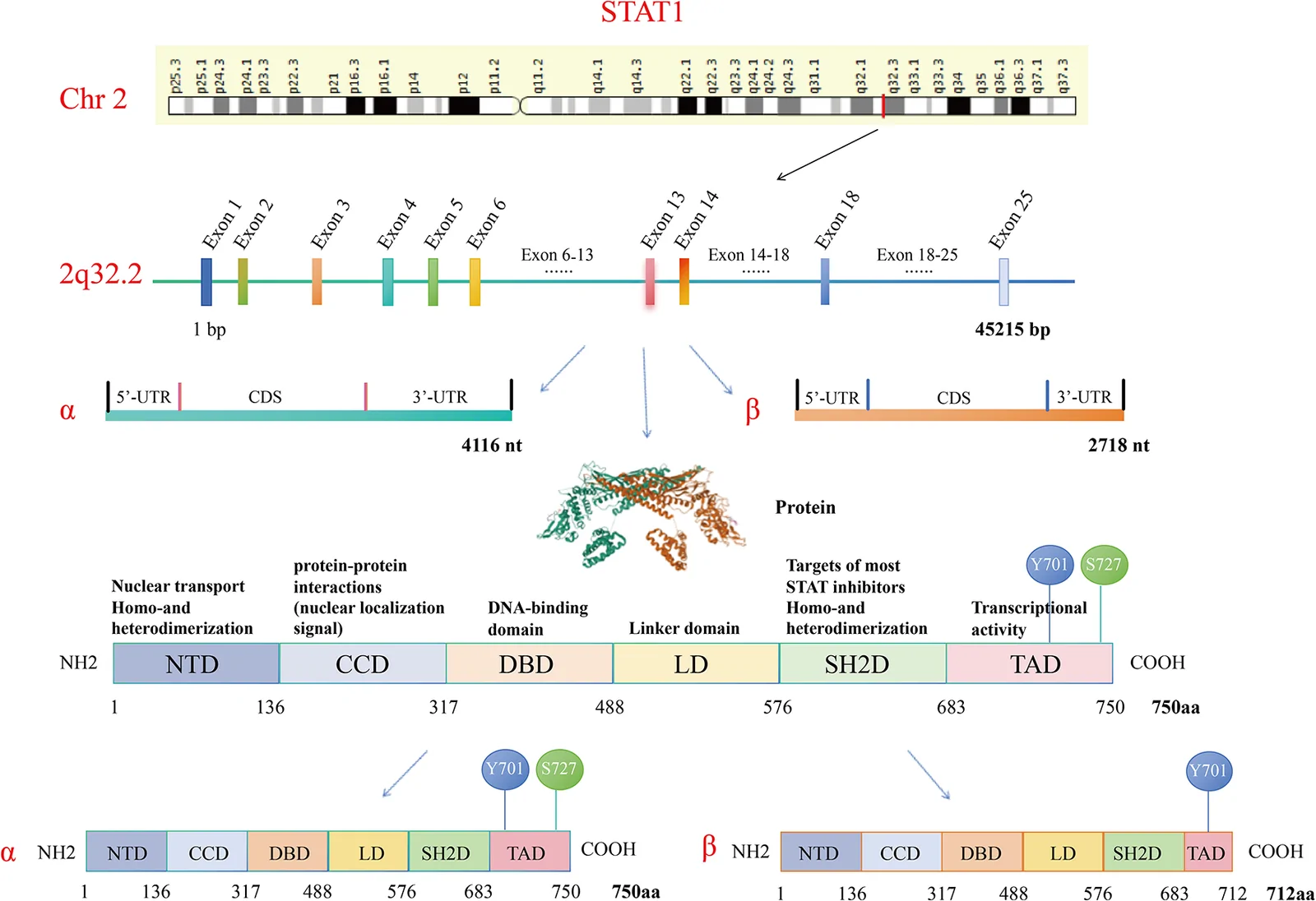
Organization of the STAT1 gene, transcripts, and isoforms of the protein. The STAT1 gene, located at 2q32.2 (45,215 bp), encodes two major isoforms: STAT1a (25 exons) and STAT1b (23 exons). Their respective mRNAs measure 4,116 bp and 2,718 bp, translating into 750- and 712-amino acid proteins. Both isoforms share key functional domains, including DNA-binding and SH2 domains essential for signal transduction. Critical phosphorylation sites at tyrosine 701 (Y701) and serine 727 (S727) regulate STAT1 activation and nuclear translocation, enabling its role in interferon signaling and transcriptional regulation. Adapted from Li et al. (100). These molecular features underlie STAT1’s pivotal functions in immune responses and cellular signaling pathways. ND, N-terminal domain; TAD, transactivation domain; CCD, coiled-coil domain; LD, linker domain; DBD, DNA binding domain; SH2D, Src homology-2 domain.

**Table 1:** Activators of STAT1 signaling, downstream targets, biological effects, and relevance to GC.

Activating category	Specific activators	Downstream targets	Biological effects	GC relevance
Type I IFN	IFN-α, IFN-β	ISGs, IRF7, MxA, OAS	Antiviral defense, apoptosis	Anti-tumor immunity; ISGF3 complex formation
Type II IFN	IFN-γ	IRF1, CXCL10, iNOS	Antimicrobial activity, Th1 polarization	Key mediator in *H. Pylori* induced gastritis
Type III IFN	IFN-λ1–λ4	Mucosal antiviral defense	Mucosal antiviral defense	Epithelial specific Immune responses
Cytokines	IL-6, IL-11,IL-27,TNF-α	SOCS3, IRF1, c-Myc	Inflammation, cell survival	Chronic inflammatory signaling in TME
Growth factors	TGF-β, EGF, PDGF,SCF	p21, p15, Smads	Growth inhibition, differentiation	Epithelialmesenchymal transition

Type II IFN (IFN-γ) and IL-27 signaling induce STAT1 homodimerization, forming gamma-activated factor (GAF) complexes that translocate to the nucleus and bind gamma-IFN-activated sequence (GAS) elements to regulate target gene transcription (40). In contrast, type I IFN (IFN-α/β) stimulation promotes the formation of STAT1–STAT2 heterodimers, which associate with IFN regulatory factor 9 (IRF9) to form the IFN-stimulated gene factor 3 (ISGF3) complex; ISGF3 binds IFN-stimulated response elements (ISREs) in target gene promoters (41–45). The transcriptional activation causes dephosphorylation of STAT1 and its recycling into the cytoplasm, thus closing the signaling loop (**Figure 2**). STAT1 facilitates pathogen defense, growth inhibition, and apoptosis. Type II IFN signaling offers defense against intracellular pathogens, such as *Mycobacterium bovis BCG* and *Salmonella* spp., and type I IFN offers antiviral defense (46). The JAK/STAT1 pathway is also responsive to *H. pylori*-infected murine models and cancer-associated fibroblasts, proving it to be relevant to gastric carcinogenesis (47).

**Figure 2:**
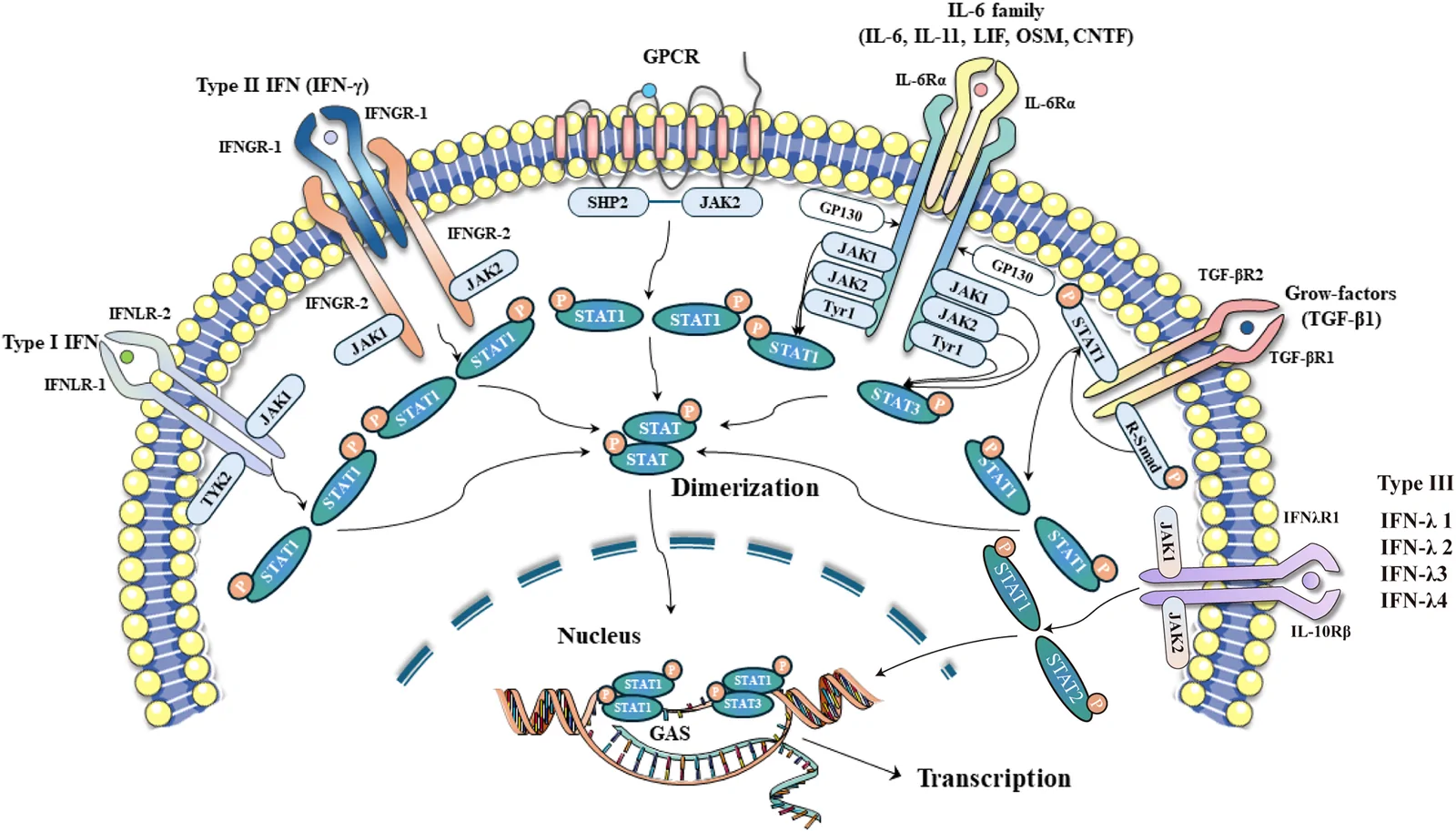
Diagrammatic illustration of type I, type II and type III interferon and cytokines signaling routes. When type I, type II and type III IFNs and IL-6 family cytokines bind to their respective receptors, these cytokines activate JAKs, which phosphorylate STAT proteins. Dimerizated STAT proteins are translocated into the nucleus and bind to specific DNA sequences to regulate gene transcription. This network highlights the intricate interactions involved in immune responses and cellular communication. Based on the Akbarin et al. (101).

## 3 Regulation mechanisms of STAT1 in GC

### 3.1 The bidirectional regulatory functions of STAT1 on tumors

STAT1 has been known to be a tumor suppressor in various types of cancer, such as GC. Constitutively active STAT1 (STAT1C) increases expression and transcription of pro-apoptotic and cell-cycle regulatory genes, such as caspases, B-cell CLL/lymphoma-2 (Bcl-2), B-cell CLL/lymphoma-XL (Bcl-XL), cyclin-dependent kinase inhibitor p27 (p27) and cyclin-dependent kinase inhibitor p21 (p21) (**Figure 3**) (48). In line with this role, STAT1 knockout mice are more susceptible to experimentally induced tumors and spontaneously develop mammary adenocarcinomas and ovarian teratomas (49, 50). STAT1 regulates multiple signaling pathways and their target molecules in both GC and *H. pylori* infected GC, thereby influencing the biological functions of GC cells (**Table 2**). Gastric proteomics has shown that the overexpression of CKLF-like MARVEL transmembrane domain-containing 4 (CMTM4) in GC cells greatly increases the activation of the STAT1 signaling, and this activation suppresses cell proliferation, migration, and invasion (51). On the other hand, CMTM4 knockdown reversed the effects, validating that CMTM4 has tumor-suppressive effects via the STAT1 signaling pathway (51). Moreover, bioinformatics studies have shown that miR-584 binds to STAT1 transcripts, and a high negative correlation is found in TCGA RNA sequencing (RNA-seq) results (52). Interestingly, miR-584 is initially increased in the early phases of primary tumor development but decreases as the disease advances, indicating a dynamic regulatory interaction that regulates tumor suppressive activities of STAT1 at different disease stages (52).

**Figure 3:**
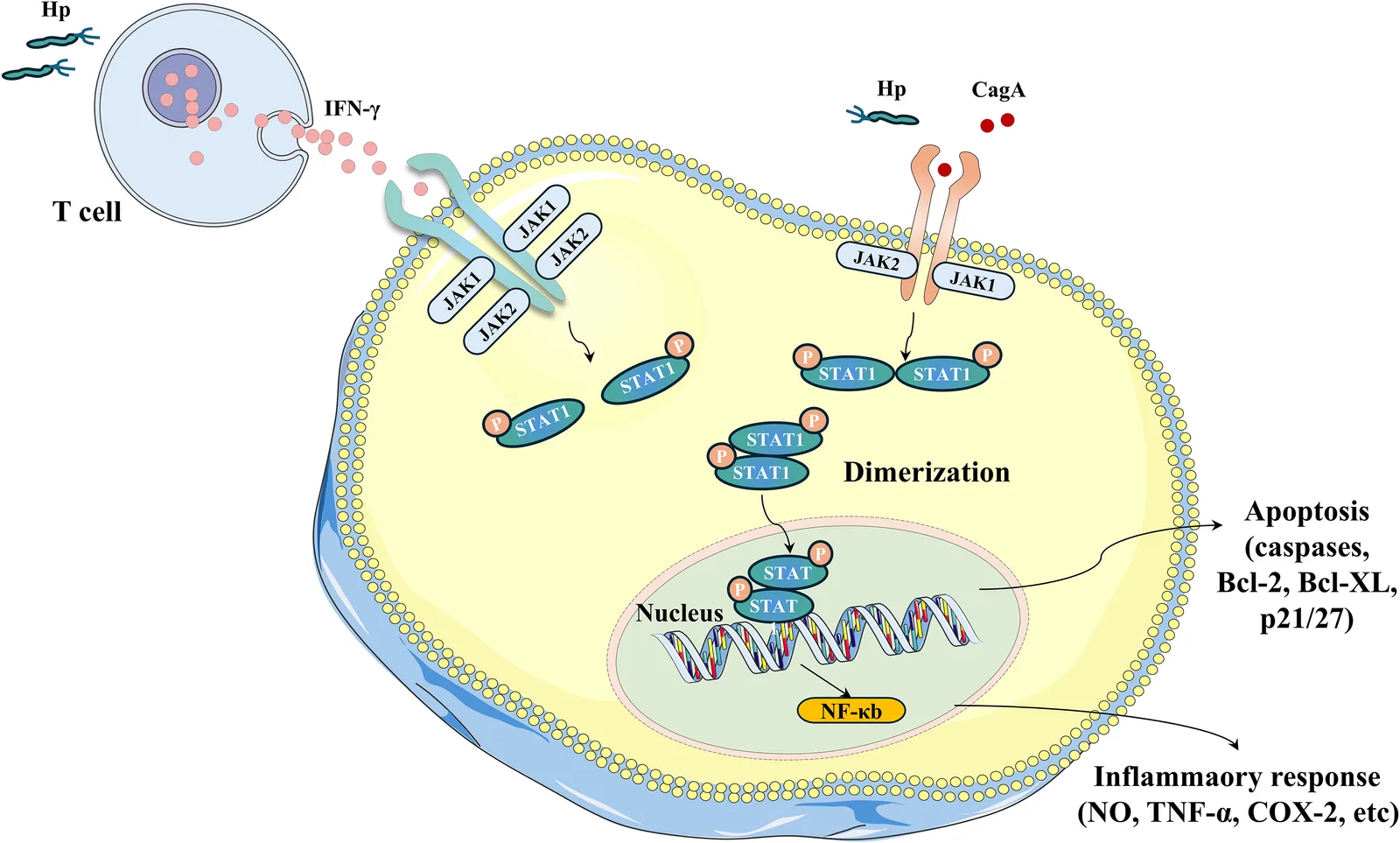
The diagram depicts the activation of JAK/STAT signaling pathway regulated by IFN-γ derived from *H. pylori* infected T lymphocytes. Upon interaction with its receptor, IFN-γ activates Janus kinases (JAKs), leading to the phosphorylation and dimerization of STAT1 proteins. These dimers translocate into the nucleus, facilitating the transcription of genes involved in apoptosis and inflammatory responses. This signaling network is crucial for immune regulation and response to pathogens like *H. pylori*.

**Table 2:** STAT1-regulated targets, biological functions, and cellular contexts in GC.

No.	Target	Biological function	Cell type	Ref.
1	CMTM4	Inhibits proliferation, migration, and invasion	GC cells	(51)
2	miR-584	Regulates tumor development and progression	GC cells	(52)
3	SHP2	Mediates immune evasion via STAT1 dephosphorylation	Macrophages	(73)
4	IL-21	Modulates Th1/Th17 responses during chronic infection	T cells (mice)	(74)
5	CCL3	Promotes inflammatory response in gastric mucosa	Macrophages	(75)
6	ASK1/MAPKs	Regulates immune response and epithelial homeostasis	Macrophages/epithelial cells	(76)
7	SLFN4	Drives MDSC development and gastric intestinal metaplasia	Gastric epithelial cells	(68)
8	PD-L1	Promotes immune evasion and GC progression	Gastric epithelial cells	(56)
9	VCAM1	Facilitates tumor invasion and poor prognosis	Cancer-associated fibroblasts	(77)
10	SMAD7	Mediates anti-inflammatory effects via TGF-β1 pathway	Gastric epithelial cells	(84)
11	OPN	Modulates apoptosis and GC development	Gastric epithelial cells	(91)

Although it is a tumor-suppressive gene, there is mounting evidence that, in certain circumstances, STAT1 can actually stimulate tumor development. STAT1 induces the expression of pro-inflammatory mediators, including TNF-α, cyclooxygenase-2 (COX-2), and nitric oxide synthase (NOS), which may exert tumorigenic effects through sustained inflammatory signaling (**Figure 3**) (53–55). Proteomic studies have shown that the expression of STAT1 gradually rises as precancerous lesions develop into invasive GC in human cohorts with gastric lesions (56). Furthermore, high levels of STAT1 are associated with high tumor stage and worse clinical prognosis, and it is possible that it contributes to the progression of the disease and resistance to therapy (56). PD-L1 on tumor cells is also upregulated by STAT1, which allows the tumor cells to evade the immune system in the TME (56).

### 3.2 Regulatory mechanisms governing STAT1 activity

Various regulatory mechanisms control the functional performance of STAT1 in GC: (a) proteasomal degradation of STAT1 protein (57); (b) epigenetic suppression of promoter by promoter methylation (58); (c) aberrant activation of negative regulators, including suppressor of cytokine signaling (SOCS) proteins and protein inhibitor of activated STAT (PIAS) family members (37); and (d) constitutive activation of STAT3, which induces cross-regulatory control over the functional performance of STAT1 (37, 59).

*H. pylori*-driven gastric carcinogenesis takes the form of chronic gastritis, through IM to GC, with a continuum of inflammatory signaling (60). *H. pylori* activates a variety of pro-inflammatory pathways that support the recruitment of immune cells in the gastric mucosa (61). Among these, IFN-γ produced by T cells is a principal mediator, activating STAT1 through JAK1/JAK2-mediated phosphorylation at Y701 following IFN-γ receptor homodimerization (62). Phosphorylated STAT1 homodimers relocate to the nucleus and bind GAS elements to induce expression of target genes that regulate cellular homeostasis and immune response (63). *H. pylori* has been characterized to have effects on the expression of STAT1 in epithelial cells. A number of studies have shown reduced STAT1 activation in cells infected with the strains of *H. pylori* that have intact type IV secretion system (T4SS) and functional CagA (64).

### 3.3 Crosstalk between STAT1 and STAT3 in GC

The interaction of STAT1/STAT3 plays a central role in the disease progression in *H. pylori*-related GC. STAT1 and STAT3 often have opposite biological functions: STAT1 typically leads to apoptosis and anti-tumor immunity, but STAT3 leads to cell survival, cell proliferation, and immunosuppression (29, 59). In acute infection, STAT1 is dominant, but in chronic infection, *H. pylori* virulence factors, especially CagA, increasingly inhibit STAT1 signaling and activate STAT3 using IL-6 and IL-10 signaling (65–67). This STAT1 to STAT3 alternation provides a permissive condition to tumor growth by concomitantly inactivating antitumor immunity and activating prosurvival genes (29).

### 3.4 Reconciling the dual role of STAT1: a context-dependent framework

The seemingly conflicting functions of STAT1 in GC can be resolved within the context-dependent model, taking into account three important variables: disease stage, cell type, and the current cytokine environment. In the initial gastric lesions, epithelial cell activation of STAT1 induces the activation of caspase and up-regulation of p21/p27 in the damaged or pre-malignant cells, thus acting as a tumor suppressor (48). Nonetheless, persistent STAT1 signaling in the TME promotes PD-L1 expression on tumor cells and myeloid-derived suppressor cells (MDSC) growth in order to evade immunity in advanced GC (56, 68). Moreover, cell-type selectivity is essential: Activation of STAT1 in cytotoxic T lymphocytes and natural killer (NK) cells improves the anti-tumor immunity, and activation of STAT1 in tumor-associated macrophages can promote immunosuppressive phenotypes by producing chronic inflammatory mediators (20, 23). This situation-specific model highlights the paramount role of time, space, and cell-type-specific factors in designing the STAT1-directed therapeutic approaches to *H. pylori*-related GC.

## 4 Molecular mechanisms of STAT1 signaling in *H. pylori* infection

### 4.1 STAT1-mediated immune responses and immune evasion

In macrophages, IFN-γ activates STAT1 to induce NO production, a critical component of the innate immune defense against intracellular pathogens (69). However, in *H. pylori*-induced gastric inflammation, IFN-γ exhibits dual functionality, contributing to both pathogen clearance and tissue damage, with SHP2 serving as a major negative regulator of the IFN-γ/STAT1 signaling axis (70, 71). The wild-type *H. pylori* strain downregulates STAT1 activation in IFN-γ-treated macrophages through outer membrane protein 18 (Omp18), whereas the Δomp18 mutant strain fails to suppress STAT1, indicating that Omp18 plays a crucial role in evading IFN-γ-mediated immunity and facilitating persistent colonization (72).

*H. pylori* infection, in concert with its CagA protein, inhibits IFN-γ-activated STAT1 through SHP2-mediated dephosphorylation, conferring resistance to IFN-γ signaling (73). Additionally, *H. pylori* disrupts STAT1-mediated IFN-γ signal transduction in epithelial cells (56). All these results support the idea that *H. pylori* uses a well-organized immune evasion response to interfere with IFN- gamma signaling through a coordinated action of CagA and SHP2 (**Figure 4a**).

**Figure 4:**
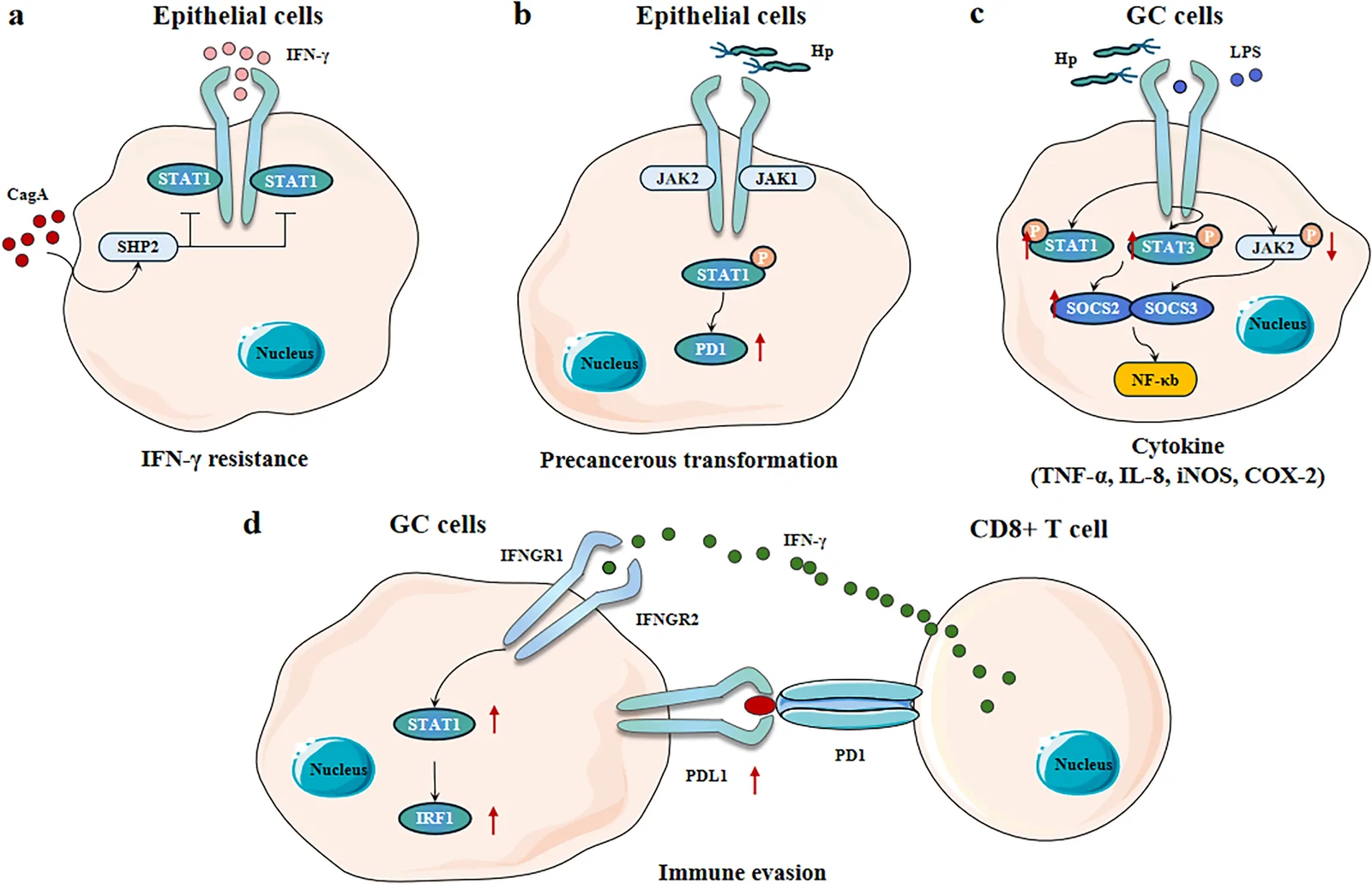
The diagram summarizes the immune mechanisms and responses in epithelial and GC cells influenced by *H. pylori*. **(a)** Epithelial cells exhibit resistance to IFN-γ, which is mediated by the interaction of STAT proteins and the recruitment of SHP2. **(b, c)** In epithelial cells or GC cells, JAK/STAT signaling promotes cytokine production and the expression of inhibitory molecules like PDL1, aiding immune evasion. **(d)** CD8+ T cells are affected by these interactions, further illustrating the complex interplay between immune responses and oncogenic transformations in the presence of *H. pylori*.

### 4.2 Signaling molecule-mediated STAT1 activation and inflammatory signaling

The activation of the STAT signaling pathway by inflammatory cytokines is a key factor in the development of *H. pylori*-associated GC (65). The host genetic reactions lead to the release of IL-6 and IL-10, which activate JAK/STAT to stimulate both proliferation and inflammation (66, 67). In chronically *H. pylori*-infected IL-21-deficient mice, upregulation of T-bet and RORγt, together with phosphorylation of STAT3 and STAT1, suggests that IL-21 modulates IL-10 levels and regulates Th1 and Th17 effector responses in a STAT1/3-dependent manner, thereby controlling *H. pylori* infection and gastritis (74).

In GC cells infected with *H. pylori*, cholesterol glycosyltransferase (CGT)-dependent cholesterol depletion disrupts lipid rafts, reducing JAK and STAT1 phosphorylation and inhibiting IFN-γ-induced signaling (64). Furthermore, *H. pylori* infection suppresses signaling from IFN-β, IL-6, and IL-22, resulting in decreased activity of JAK/STAT-dependent genes (64). Wei et al. showed that STAT1 is a transcription factor of CCL3, and that *H. pylori* induces macrophages to release CCL3 by the JAK/STAT1 pathway (75). Hayakawa et al. have shown that in the infection of *H. pylori*. ASK1 knockout mice, enhanced STAT1 and NF-κB activation driven by elevated IL-1β from apoptotic macrophages promoted Th1-polarized responses (76). Additionally, *H. pylori* infection increases IFN-α and Schlafen 4 (SLFN4) expression and activates the JAK2/STAT1 pathway in bone marrow cells; IFN-α triggers nuclear translocation of STAT1 and drives SLFN4^+^ MDSC development, contributing to gastric intestinal metaplasia (68).

In GC, STAT1 and its target gene PD-L1 are immensely up-regulated. *In vitro* experiments indicate that *H. pylori* infection increases the activation of STAT1 and expression of PD-L1 in gastric epithelial cells (56). This PD-L1, induced by STAT1, inhibits immune surveillance in the gastric mucosa and facilitates the progression of GC out of premalignant lesions (**Figure 4b**) (56). Moreover, the *H. pylori* infection facilitates tumor progression and poor prognosis due to cancer-associated fibroblast (CAF)-produced vascular cell adhesion molecule 1 (VCAM1) that is conditional on JAK/STAT1 signaling and which might be a future therapeutic target (77).

### 4.3 Integrative conceptual model: from infection to carcinogenesis

A temporal model of *H. pylori*-mediated STAT1 manipulation can be proposed. During acute infection, *H. pylori* triggers robust STAT1 activation through IFN-γ signaling, promoting antimicrobial gene expression and inflammatory cytokine production. As infection advances, *H. pylori* virulence factors, especially CagA and CGT, suppress the activation of STAT1 by increasingly dephosphorylating lipid rafts with SHP2 and depleting cholesterol of lipid rafts via dephosphorylation and cholesterol depletion, respectively (64, 73). This attenuation of STAT1, coupled with the mutual activation of STAT3, forms an immunosuppressive environment that promotes epithelial conversion. Possible residual STAT1 activity in advanced gastric lesions paradoxically leads to immune escape with PD-L1 upregulation and MDSC expansion (56, 68). This model provides a framework for understanding the immunity-to-carcinogenesis transition and identifies intervention points (**Table 3**).

**Table 3:** Integrative conceptual model of STAT1 manipulation by *H. pylori* during the transition from infection to carcinogenesis.

	Phase 1: acute infection	Phase 2: chronic infection	Phase 3: advanced GC
Correa stage	Normal mucosa → Chronic gastritis	Atrophic gastritis → Intestinal metaplasia	Dysplasia → Gastric cancer
STAT1 status	HIGH: IFN-γ → JAK1/JAK2 → p-STAT1 (Y701) → GAF → GAS elements	SUPPRESSED: CagA → SHP2 dephosphorylation; CGT → lipid raft disruption	PARADOXICAL: Residual STAT1 drives PD-L1 and SLFN4^+^ MDSC expansion
STAT3 status	Low	Rising: IL-6/IL-10 signaling drives STAT1→STAT3 switch	Dominant: Pro-survival and proliferative gene expression
Immune profile	M1 macrophages; ROS, NO, TNF-α, IL-6, IL-8; Th1 polarization	M1 → M2 polarization; immunosuppressive cytokine milieu	PD-L1^+^ tumour cells suppress CD8^+^ T cells; MDSC accumulation; CAF-derived VCAM1
Key virulence mechanisms	*H. pylori* triggers PRR activation and IFN-γ production by T cells	CagA (via T4SS) activates SHP2; CGT depletes cholesterol from lipid rafts	Sustained CagA/SHP2 suppression; chronic inflammation drives epithelial transformation
Net biological effect	Host defense: Antimicrobial gene expression, apoptosis of infected cells (tumour suppression)	Immune evasion: Attenuated IFN-γ signaling permits persistent colonisation	Tumour promotion: Immune escape via PD-L1; proliferation via STAT3; invasion via VCAM1
Therapeutic intervention points	Maintain STAT1 activation; support innate immunity	Restore STAT1: Probiotics (L. acidophilus, L. plantarum); JAK modulators; eradication therapy	Anti-PD-1/PD-L1 + ruxolitinib; napabucasin (STAT3); fludarabine (STAT1); nanoparticle delivery; CAR-T

## 5 Therapeutic strategies targeting STAT1 signaling in GC

Treatment of *H. pylori* infection remains a significant challenge, as it is a major contributor to chronic atrophic gastritis, peptic ulcers, and a well-established risk factor for GC (78, 79). Although eradication therapy provides benefits for patients with peptic ulcer disease or mucosa-associated lymphoid tissue (MALT), evidence regarding the clinical benefits of eradication in GC prevention remains limited (80, 81). Therefore, reducing the inflammatory response associated with *H. pylori* infection represents a prudent approach to preventing *H. pylori*-related malignancy (82).

### 5.1 Probiotic-mediated STAT1 modulation

Pretreatment with probiotics, including Lactobacillus plantarum, Lactobacillus rhamnosus, and Lactobacillus acidophilus, effectively reduces *H. pylori*-induced inflammatory mediators (TNF-α, IL-8, iNOS, and COX-2) by blocking NF-κB nuclear translocation (**Figure 4c**) (83). The anti-inflammatory effects of probiotics involve SOCS-2/3 by activating the phosphorylation of STAT1/3 or suppressing the activation of JAK2, suggesting that activating STAT1/3 can mediate the effect of probiotics (83). Furthermore, *Lactobacillus acidophilus* pretreatment reduces *H. pylori*-induced IL-8 expression by inactivating JAK1 and STAT1, potentially activating the TGF-β1/Sma-and-Mad-related protein (SMAD) pathway, and mitigates inflammation by decreasing SMAD7 transcription and nuclear nuclear factor-κB (NF-κB) production (84).

### 5.2 PD-1/PD-L1 axis and immune checkpoint modulation

PD-1 and PD-L1 expression is regulated by IFN-γ-mediated JAK/STAT signaling, with STAT1 and STAT3 pathways uniquely involved (85, 86). Inhibiting this signaling axis is a possible way to reestablish immune balance by decreasing PD-L1 on the tumor cells and immune cells in the TME (**Figure 4d**) (87). SiRNA knockdown of either STAT1 or STAT3 is effective in inhibiting cytokine-induced PD-L1 expression in human monocytes and tumor cells (88). Additionally, selective blocking of the JAK2/STAT1 and JAK2/STAT3 pathways by using JAK inhibitors, including ruxolitinib, could benefit the effectiveness of anti-PD-1 immunotherapy (89).

The mice with STAT1 knockout show a decreased response to chemokine (C-X-C motif) ligand-10 (CXCL10) and are more susceptible to *H. pylori* infection (90). The inhibition of osteopontin (OPN) stimulates gastric carcinogenesis caused by *H. pylori* via STAT1 signaling, which shows that OPN regulates the inhibition of the *H. pylori*-induced gastric carcinogenesis (91). It has been established that both the IFN-β–STAT3 and IFN-γ–STAT1 pathways play critical roles in regulating PD-L1 expression (92). A peptide mixture from Kang Fuxin (PEEPA) modulates extracellular regulated protein kinases (ERK) and STAT1 signaling, promoting gastric epithelial regeneration in atrophic gastritis (93).

### 5.3 Small-molecule STAT inhibitors and targeted delivery

Selective JAK1/JAK2 inhibitor (ruxolitinib) has been shown to lower the phosphorylation of STAT1 and STAT3 in preclinical GC models (89). Initially a product of antimetabolite development, Fludarabine is a selective inhibitor of STAT1 activation and has been demonstrated to potentially regulate anti-tumor immunity. Further, napabucasin (BBI-608) is a STAT3-targeted drug that can indirectly re-establish tumor-suppressive activity of STAT1 by destabilizing the STAT3-dominating signaling equilibrium. Nevertheless, these agents have yet to be translated into clinical applications because of the fear of target specificity, off-target immunosuppression, and dose-limiting toxicities that could weaken host immunity.

Targeted drug delivery systems with nanoparticles and antibody-drug conjugates can also be used to improve efficacy and reduce systemic toxicity in *H. pylori*-associated GC.

### 5.4 Combination immunotherapy strategies

The rational approach is to combine the modulation of the STAT1 with immune checkpoint inhibitors, since JAK/STAT inhibition can predispose tumors to the anti-PD-1/PD-L1 treatment (89). Possible combinations with chimeric antigen receptor (CAR)-T cell therapy, cancer vaccines, and bispecific antibodies should also be investigated, as STAT1 is a central point in anti-tumor immunity, as it simultaneously controls antigen presentation, T cell activation, and immune checkpoint expression.

### 5.5 Clinical trials and current limitations

A number of clinical studies are in progress testing JAK/STAT pathway modulators in gastrointestinal cancer, such as ruxolitinib with capecitabine in advanced GC and tofacitinib in inflammatory gastrointestinal diseases, but few directly test STAT1 in *H. pylori*-associated GC. Major obstacles persist, such as selective immunological targeting of STAT1 without disturbing the normal functioning of the immune system, paradoxical tumor promotion, and no validated patient-stratifying companion biomarkers.

At present, *H. pylori* infection management still relies on conventional antibiotic-based eradication regimens, including triple and quadruple therapy. However, rising antibiotic resistance, adverse effects of prolonged multi-drug regimens, and the substantial financial burden on patients underscore the urgent need for novel therapeutic approaches.

## 6 STAT1 as a potential biomarker in GC

A growing amount of evidence indicates that the expression of STAT1 is intimately linked to microbial infections and can be used as a potential diagnostic tool (**Figure 5**) (94, 95). Yi et al. discovered that a potential biomarker of active tuberculosis using gene expression omnibus (GEO) data includes STAT1 (96), and later findings suggest that STAT1 can also be used as an indicator of immune infiltration processes after anti-tuberculosis therapy (97).STAT1 also could act as an immunotherapy biomarker. Zemek et al. noted that the gene expression profile of immune checkpoint-inhibitor (ICI)-responsive tumors is marked by increased expression of STAT1 and an inflammatory signature (98). Zhang et al. showed that a combination of functional testing (phosphorylation levels) and DNA sequencing of STAT1 may be an efficient method to diagnose STAT1 gain-of-function (GOF) mutations (99). Li et al. have reported that the p-STAT1 and PD-L1 levels are correlated with immune infiltration and proliferation in tissues and that their up-regulation is associated with poor prognosis in the Cancer Genome Atlas (TCGA) stomach adenocarcinoma (STAD) database (56).

**Figure 5:**
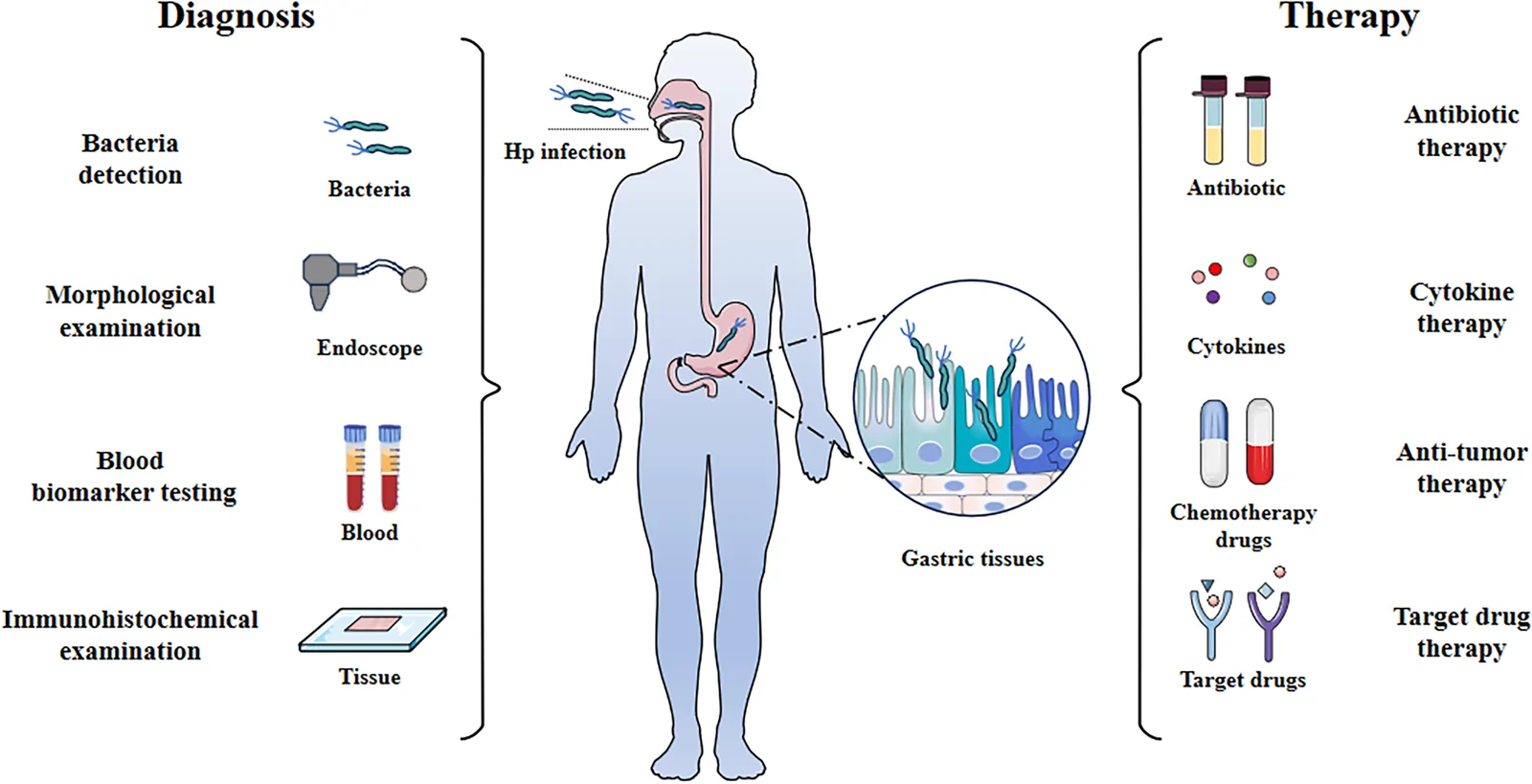
Schematic diagram of diagnosis and treatment strategies for GC infected with *H. pylori*. The diagnosis of GC infected with *H. pylori* has become dependent on gastroscopy, bacteriology, blood markers, and pathological tissue examination. In addition, the therapy could be achieved through antibiotics, immune factors, and chemotherapy drugs.

Although these results are encouraging, one must note that the existing evidence of the use of STAT1 as a clinical biomarker of GC is mostly experimental, bioinformatic, and preclinical in nature. There are no clinical validation studies that evaluate the sensitivity, specificity, and positive predictive value of STAT1 in future GC cohorts. Diagnostic performance of STAT1 has not been specifically compared to known GC biomarkers, such as human epidermal growth factor receptor 2 (HER2) overexpression, PD-L1 combined positive score (CPS), microsatellite instability (MSI) status, or Epstein-Barr virus (EBV) positivity, all of which have shown clinical utility in informing therapeutic decisions.

Moreover, the expression of STAT1 is not tumor-specific; many physiological and pathological conditions, such as viral infections, autoimmune diseases, and systemic inflammation, regulate it, which may reduce its specificity as a GC biomarker. Future research needs to consider determining the expression or phosphorylation of STAT1 in parallel with other complementary biomarkers in order to generate multianalyte panels with greater diagnostic sensitivity. The evaluation of STAT1 in liquid biopsies such as circulating tumor DNA (ctDNA) and extracellular vesicles is a new field that can improve clinical feasibility and access.

Currently, the detection of *H. pylori* infection and associated diseases is based on the traditional endoscopy and bacteriological test. Evaluation of the risk of infection by *H. pylori* based on the targeting of the expression of the STAT1 gene or protein in blood and tissues is a potentially valuable diagnostic approach. Nevertheless, the relative lack of studies that directly prove the clinical utility of STAT1 in the diagnosis of GC, as well as the need to interpret results with care, makes it a matter of priority in the future to identify a simple, safe, and reliable diagnostic application.

## 7 Future prospects

STAT1 holds a pivotal role in the pathogenesis of *H. pylori*-related GC, affecting disease pathogenesis, treatment options, and diagnostic methods. Being an initiator of immune response and inflammation, IFN-γ activates STAT1, which enhances antimicrobial action in addition to causing chronic inflammation that may lead to the transformation of gastric epithelial cells. This duality underlines the intricacy of STAT1’s role in carcinogenesis and the importance of delicate therapeutic solutions.

### 7.1 Emerging technologies and research directions

A number of new technologies have great potential to advance our knowledge of STAT1 in the *H. pylori*-associated GC. Single-cell transcriptomics has already started to disclose the cellular heterogeneity of STAT1 expression in the gastric mucosa and TME, allowing the identification of distinct cell types in which STAT1 mediates protective or pathogenic responses (61). Spatial transcriptomics provides the possibility to map STAT1 signaling throughout the Correa cascade, giving a view of the spatial dynamics of immune regulation.

The characterization of the multi-omics TME will be fundamental to comprehending the role of STAT1 interactions in influencing the immunological context of a gastric tissue infected with *H. pylori*. Specifically, a single-cell resolution definition of the STAT1/STAT3 balance of the TME can be used to discover the targeted vulnerabilities that can be therapeutically exploited.

### 7.2 Artificial intelligence-assisted biomarker discovery

Using artificial intelligence (AI) and machine learning to discover biomarkers is a fast-changing frontier. Multi-dimensional datasets, such as genomic, transcriptomic, proteomic, and clinical data, can be utilized based on AI-based algorithms to determine the predictive value of STAT1 expression signature as a predictor of disease progression and response to treatment. Histopathological images processed with deep learning can potentially support automated evaluation of STAT1 activation, which can mitigate the subjectivity of diagnosis.

### 7.3 Therapeutic outlook

The implementation of strategies that would repair STAT1 tumor-suppressive functions in early disease and prevent pro-tumorigenic functions in advanced GC is an ideal but challenging goal. It is possible to combine STAT1 modulation with immune checkpoint inhibitors to enhance treatment outcomes in patients infected with *H. pylori* due to the ability to address the tumor-specific and immunological mechanisms of the disease course. Precise therapeutic interventions based on the specific patient profile could potentially be achieved in the future by developing cell-type-specific STAT1 modulators based on the understanding of single-cell and spatial transcriptomic studies.

The level of phosphorylation and expression of STAT1 can be used to determine people with an increased risk of GC due to *H. pylori* infection. The addition of STAT1 to multianalyte panels stratified by AI could enable early detection. All in all, STAT1 is an encouraging but complicated target for further development of GC treatment in the context of *H. pylori* infection, and its full therapeutic and diagnostic capabilities are still to be clarified with the help of thorough translational and clinical research.
